# Nutraceuticals in Periodontal Health: A Systematic Review on the Role of Vitamins in Periodontal Health Maintenance

**DOI:** 10.3390/molecules23051226

**Published:** 2018-05-20

**Authors:** Alfonso Varela-López, María D. Navarro-Hortal, Francesca Giampieri, Pedro Bullón, Maurizio Battino, José L. Quiles

**Affiliations:** 1 Dipartimento di Scienze Cliniche Specialistiche ed Odontostomatologiche (DISCO)-Sez. Biochimica, Facoltà di Medicina, Università Politecnica delle Marche, 60131 Ancona, Italy; avarelalopez@gmail.com (A.V.-L.); f.giampieri@univpm.it (F.G.); m.a.battino@univpm.it (M.B.); 2 Department of Physiology, Institute of Nutrition and Food Technology “Jose Mataix”, Biomedical Research Center, University of Granada, Avda. Conocimiento s/n, 18100 Armilla, Granada, Spain; mdnavarrohortal@correo.ugr.es; 3 Department of Stomalogy, Dental School, University of Sevilla, C/Avicena s.n., 41009 Sevilla, Spain; pbullon@us.es

**Keywords:** diet, gingivitis, micronutrients, nutrition, oral health, periodontitis, supplementation

## Abstract

Periodontal disease, a relevant public health problem worldwide, is generally considered a common pathology of elderly people. In this respect, there is agreement about that nutritional status may be a modifying factor in the progression and healing of the periodontal tissues. Vitamins have been recommended as nutraceuticals for prevention and treatment of some pathological conditions, such as cardiovascular diseases, obesity or cancer. Thus, a systematic approach to determining how the different vitamin type could ameliorate periodontal risks or improve periodontal health is necessary to further the understanding of the potential benefits and risks of vitamins supplementation use. For this, a systematic review of English-written literature in PubMed until February 2018, which included both human and animal research on the relationship of each vitamin with periodontal disease, was conducted. Among all the analyzed vitamins those with antioxidant capacity and effects on immune system seem to be useful for prevention or improvement of periodontal disease, as well as those implicated in bone metabolism. In the first case, there are quite information in favor of various vitamins, mainly vitamin C, that is the most studied. In the second case, vitamin D seems to have the most relevant role.

## 1. Introduction

Periodontal disease, a relevant public health problem worldwide, is generally considered a common pathology of elderly people [1]. In the United States (US), its prevalence is over 47%, with 64% of adults aged 65 years and older having either moderate or severe periodontitis [2]. It is essentially a chronic inflammatory disease caused by specific oral microorganisms (e.g., *Porphyromonas gingivalis* and *Aggregatibacter actinomycetemcomitans*), characterized by the loss of supporting periodontal ligament and alveolar bone and is the first cause of tooth loss [3]. Periodontal disease is influenced by many risk factors, such as alcohol, stress, smoking, genetics, diabetes, and hormonal alteration status (i.e., pregnancy or menopause) [4], so that the maintenance of oral and periodontal health becomes today a challenge, especially considering that periodontal disease can be itself a risk factor for a number of chronic disorders. Indeed, it is generally accepted that periodontitis is strictly associated with many chronic diseases, such as type 2 diabetes mellitus (DM2), cardiovascular diseases, inflammatory bowel disease and rheumatoid arthritis [5]. Therefore, there is a growing interest in the relationship between oral health and systemic health.

Undoubtedly, oral microorganisms are indispensable for the pathogenesis of periodontal disease, but, among risk factors, nutrition represents an influential aspect that is often neglected [1]. Indeed, nutritional factors have a vital importance for the equilibrium between oral microorganisms and the host response, from which depends the onset and progression of periodontal disease [6,7]. In the past, health-focused dietary approaches have mostly focused in reducing the consumption of “undesirable” dietary components such as sodium, refined sugars, and saturated fats. However, attention on promoting the consumption of “curative nutrients” has been expanded. This perception of “food or nutrient as a medicament” has encouraged the search of a growing number of new substances categorized from dietary supplements to “nutraceuticals”, with the aim of improving human health, making us fitter and more resilient to disease [8,9]. “Nutraceuticals” are a category of substances without a legal definition, often sold as dietary supplements or components of conventional foods [10]. Zeisel [11] defined nutraceuticals as dietary supplements that delivers a concentrated form of a biologically active component of food in a non-food matrix to enhance health. A dietary supplement is a product that is intended to supplement the diet that bears or contains one or more ingredients like, vitamin, mineral, an herb, an amino acid or a concentrate, metabolite, constituent, extract, or combinations of these.

Because the scientific characterization and commercial regulation of such substances vary from country to country [8,11,12], their popularity imposes the medical and scientific community a constant revision of their therapeutic potentials. Nutritional status of the host has been widely recognized as a possible promoting factor in many inflammatory conditions such as periodontal diseases [13,14]. Actually, the consumption of specific foods or extracts of specific foods have been shown to have an effect on both the oral health and the general health of patients with periodontal disease [6,7,15]. Among dietary factors, those nutrients that exert antioxidant and immunomodulatory effects [6,7,16,17] or are implicated in bone metabolism [6,7,18] could be a promising candidate for the prevention or the management of periodontal diseases. Vitamins that have been recommended as nutraceuticals for prevention and treatment of some pathological conditions, such as cardiovascular diseases, obesity or cancer [6,19], could have an interesting role. In fact, they are susceptible to be delivered as tablets or added to foods and a 20–30% of Americans currently consume multivitamin supplements daily, indicating high public interest in the prevention of chronic diseases through a nutrition-based approach [19].

In this context, epidemiologic studies generally evaluate foods rather than specific bioactive food components and there is still a scarcity of reports describing any effects of dietetic interventions on periodontitis. Thus, a systematic approach to determining how the different types of vitamins could ameliorate periodontal risks or improve periodontal health is necessary to understand the potential benefits and risks of vitamins supplementation. The aim of this review is to analyze the main published data until February 2018 on the relationship between vitamins and the development and progression of periodontal disease both in humans and animals.

## 2. Results and Discussion

Our initial search detected 6055 publications. Title and abstract screening left 51 potential articles according to the inclusion and exclusion criteria. Full-text screening and reading led to a final number of 41 articles which were included in this this review. All vitamins, except vitamin K, were represented in the selected literature, although several studies evaluate the effects of different vitamins role at the same time. The results from these studies concerning each vitamin, as well as their most relevant methodological aspects for the aim of this review, are presented and discussed in separated sections below.

In general, evidence supporting certain grade of relationship of vitamins with periodontal health is reduced in many cases. Most of information is provided by observational studies, particularly cross-sectional studies, and many of them were performed in specific groups. These groups differed in age, disease or health condition making thus difficult to translate these findings to the overall population. In addition to that, few articles deepened on the mechanisms under the observed relationships. Among other reasons, this could be due to methodological approach of this review. However, there are other reasons inherent to studies investigating diet or nutrition role in a pathology or disease outcomes [14]. For instance, possible relationships reported between nutritional status and disease outcomes are usually non-linear and typically exhibit a threshold effect [19]. Likewise, the methodology used to obtain data about both, dietary intakes and nutritional status, leads to other problems. However, dietary intakes estimation methods are usually susceptible to memory errors and bias [14]. Likewise, nutritional status biomarkers are not meaningful for certain nutrients [20]. Unfortunately, it was also not uncommon that dietary supplements also were ignored or normalized with the same value for all subjects. Lastly, there is also a wide variability with respect to the evaluation of periodontal health in the selected studies. Concerning this, assessed outcomes included various clinical parameters obtaining in the oral examinations (probing pocket depth (PPD), clinical attachment loss (CAL), bleeding on probing (BOP)), but also different indexes (Russell’s Periodontal Index (RPI), Community Periodontal Index (CPI) or Community Periodontal Index of Treatment Needs (CPITN), Periodontal Index (PI), or Gingival Index (GI)) as well as different disease definitions or classifications. Likewise, number and loss of teeth were taken into account, although these depend only partially due to periodontitis. Consequently, findings from the studies collected in this review have a complex interpretation. This makes difficult to draw conclusions about the role of the different vitamins in periodontal disease, particularly for those less studied.

### 2.1. Vitamin D

Vitamin D is an inclusive term for several forms of the hormone cholecalciferol whose biologically active form is 1,25-dihydroxycholecalciferol (1,25(OH)2D3) [21]. This vitamin is the second most represented nutrient among the observational studies selected in this review with a total of eight articles (Table 1). In detail, we found four cross-sectional, three case-control and two cohort studies. The best-known action of 1,25(OH)2D3 is regulation of plasma calcium and phosphorus levels affecting bone development and metabolism, but it is also essential for cell development and neuromuscular functioning [21,22]. In addition, vitamin D also has a role in the immune system and inflammation, inhibiting pro-inflammatory cytokines and T-lymphocyte proliferation, at least in vitro [23]. Actually, antigen-presenting cells, macrophages and lymphocytes express a nuclear receptor for vitamin D. Activities on both, bone and immune system, result interesting for periodontal disease prevention and management since they would be useful preserving alveolar bone and gingival tissue. In contrast with other vitamins, animals can endogenously produce vitamin D by conversion of 7-dehydrocholesterol found in the skin into pre-vitamin D after exposure to the sun [24]. Thus, sun exposure notably influences RDA [25]. For this reason, vitamin D status is best determined by the level of serum or plasma 25-hydroxyvitamin D (25(OH)D), which reflects the total contribution from both dietary and endogenous sources. Here, most of selected observational studies assessed nutritional status for this nutrient by 25(OH)D levels in blood, although dietary intakes were estimated in two of the selected cross-sectional studies.

Most interesting results were provided by a 20 year longitudinal study in the U.S. known as the Health Professionals Follow-Up Study, where predicted 25(OH)D score was inversely and dose-dependently associated with self-reported tooth loss and periodontal disease [26]. However, participant ages ranged from 40 to 75 years old. This has been supported by a cross-sectional study conducted on a sample of 11,202 subjects participating in NHANES III reported an inverse association between serum 25(OH)D and CAL in men and women, but only in subjects over 50 years old in both genders [27]. In this sense, a cross-sectional study in a male relatively aged segment (62.9 ± 7.6 years), but with a more reduced ample size (N = 562) also suggest this an inverse association between vitamin D intake and severe periodontitis according to American Academy of Periodontology (AAP) criteria. A similar association was found for moderate-to severe ABL (i.e., 40% of ABL at three or more sites) calculated from conventional periapical radiographs [28]. These studies are in consistency with the previous study and, along with it, point out adults at middle or higher age as risk group where correct vitamin D intake could have a key role in periodontal health maintenance. Similarly, a case control study with a more reduced sample of periodontitis-affected and periodontally healthy subjects from Puerto Rico has reported a associations between serum levels of 25(OH)D and periodontal condition [29]. In contrast, no significant association was found in a cross-sectional study in a representative sample of Denmark [30]. Likewise in other cross-sectional study performed on participants in fourth Korea National Health and Nutrition Examination Survey (KNHANES) evaluating association between vitamin D deficiency, in this case determined by serum levels, and prevalence of periodontitis defined by a CPI of 3 or higher in 6011 individuals [31]. However, no association was observed. On the other hand, stratified analyses revealed a significant association in current smokers [31]. Absence of association in one of these studies could be due to periodontal health measure since CAL or CPI that is based in PPD and can under evaluate bone loss. Still, this last study suggest that vitamin D could be of important for periodontal health in smokers as occurred for other vitamins.

Combined results from all previous studies suggest that starting to take vitamin D supplementation from middle age could be particularly interesting for preventing periodontal disease advancement. This was expected given the role of vitamin D in bone metabolism as well as role of age as risk factor for bone health maintenance. In this sense, several studies have been performed on subgroups with conditions that represent a risk for bone health. In relation to age, two studies were only focused on postmenopausal women but reported association depending on parameter using to evaluate periodontal health or nutritional status. These included a cohort study [32] where no significant associations were found after a 5 year follow-up period with different parameters related to periodontal health. Included parameters were alveolar crestal height (ACH), CAL, PPD and % BOP. Results did not confirm any association with 25(OH)D levels. In contrast, an inverse association was found in a cross-sectional study in which two definitions of periodontal disease, one based on ACH measures and tooth loss and the other based on CDC/AAP criteria, were used [33]. Likewise, acute oral inflammation was assessed by percentage of BOP and they found that 42% of same group showed lower odds of having 50% of gingival sites that bled or more. Lastly, pregnancy also has been taken into account in a case-control study. In this, women with clinically moderate to severe periodontal disease presented lower levels and higher prevalence of vitamin deficiency than periodontal healthy women [34]. According to these results, recommendations of vitamin D (and probably calcium)-supplements use for people under physiological or pathological conditions that increase the risk of suffers bone pathologies or pathological alterations, also could contribute to protect periodontal tissues.

Interestingly, in a case-control study that discriminated between patients with generalized aggressive periodontitis and chronic periodontitis, it was noted higher plasma calcifediol levels in patients with aggressive periodontitis compared with healthy controls, but not in chronic periodontitis cases [35]. Additionally, partial intra-groups correlations with main clinical outcomes (PPD, CAL, bleeding index, % surfaces with BOP) were analyzed and it was found a positive association with BI in this group. Confounding could be explained by the differences among these studies. The first case-control did not evaluate possible confounding effects for skin color or sun exposure (while data collected over a 6 years period) that showed differences between groups when they were analyzed in the second case besides civil status, weight, medical insurance [34]. This could lead to underestimate towards null the associations with chronic periodontitis. On the other hand, aggressive periodontitis was not considered in the third investigation and it showed inverse relationships with vitamin D compared to more common periodontal disease or chronic periodontitis, although different etiology could be responsible.

### 2.2. Vitamin A

Vitamin A is a liposoluble vitamin can be present in different forms including retinol (A_1_), retinal, 3,4-dehydroretinol (A_2_) and 3-hydroxyretinol (A_3_). However, it his known that about 50 possible carotenoids (a group lipid-soluble plant pigments) act as vitamin precursors, being β-carotene the most important. For this reason, it was also selected studies assessing levels or intake of carotenoids with provitamin A activity (mainly α- and β-carotene, and β-cryptoxanthin). Attending to that, seven publications were collected, although they contain only observational studies (Table 2), mostly cross-sectional studies. Among them, only three studies estimated dietary intakes of vitamin A. On one hand, Freeland et al. [36] reported a significant negative correlation at bivariate level between vitamin A intake and RPI in a smaller sample constituted by patients from a suburban dental clinic. Notwithstanding, these authors also analyzed the relationship with more nutrients and vitamin A intake was not considered among significant predictors in multiple analysis after using R^2^ improvement. Additionally, dietary intake data only were available from 56 of 80 patients, which could produce a selection bias that was no evaluated. On the other hand, a case-control study in female adolescent with and without gingivitis indicates absence of differences for both, intake or prevalence of marginal deficiencies of intake (below one third of recommendations) [37]. However, in the two first studies, dietary intakes were estimated by a 24 h recall and a 3 days food diary, respectively. Such information only represents current diet, which along with the possible memory errors and bias may not represent real nutritional status of the sample [20]. Anyway, most of studies have investigated possible associations of serum levels of vitamin A (or retinol) and different carotenoids with periodontal health. Results from one of these investigations are particularly significant due to it was performed with a sample of 11,480 adults from the third National Health and Nutrition Examination Survey (NHANES III), which are a representative of the United States (US) non-institutionalized population [38]. In this, significant associations with periodontitis were reported for α-, β-carotene and β-cryptoxanthin when analysis was not restricted to most severe cases of periodontitis (defined by the presence of at least one site with CAL of 4 mm or higher and PPD of at least 4 mm). Still, severe periodontitis (defined by the presence of two or more mesiobuccal sites with CAL of at least 5 mm and at least one mesiobuccal site with PPD of 4 mm or higher) was significantly associated with β-carotene and vitamin A [38]. In contrast, no significant associations with RPI was reported by the study in patients from a suburban dental clinic recently mentioned [36]. Moreover, as indicated, vitamin A intake was also not related to RPI in multiple analysis in the same study. Anyway, it is important to point out that blood samples were collected after no fasting indication in the last study. The combination of all this methodological issues along with the reduced subset of individuals evaluated would contribute to explain the absence of significant associations found in this study. In spite of most studies has been considered to evaluate vitamin A nutritional status by measurement of blood levels, there was also a study in an ethnic group from Sri Lanka that evaluated it by means of deficiency clinical symptoms presence, but no association was also found with RPI again [39]. However, in this last periodontal disease degree could be undervalued since oral examinations outside of University were made into bad conditions such as poor luminosity and non-appropriate chairs, among others. Despite these results, it is important to point out that Vitamin A is stored in the liver and serum levels are closely regulated, so low and deficient serum values are linked to depleted hepatic stores [40].

Other known risk factors have been also taken into account in many of the selected studies, especially age and smoking. Some studies have been performed only in elderly adults. These include a retrospective cohort study in 334 Japanese from Niigata city where dietary intakes of α- and β-carotene were estimated by a brief self-administered diet-history questionnaire (BDHQ) [41], and a cross-sectional in 1258 PRIME study participants (North Ireland) where serum levels of retinol, α and β-carotene, and β-cryptoxanthin were registered [42]. In spite of the first one only was focused in two carotenoids, it was noted a negative association between dietary β-carotene intake and no of teeth with periodontal disease progression, even after adjustment for several cofounders, although α-carotene intake did not show association [42]. Unfortunately, the BDHQ did not include supplements and it was self-assessed.

Notwithstanding, the role of β-carotene was supported by results from the second study that was carried out in a sample with a considerable size. This also suggests an important role for β-cryptoxanthin, as well as for α-carotene and retinol, but only when analysis were not restricted to more severe periodontitis cases. However, it is important to point out that participants were only men in the last one. Concerning smoking, in the study performed on data from NHANES III [38] only β-carotene levels maintain an inverse association with periodontitis when analysis was restricted to never-smokers. In this context, other studies adjusted OR by age or include smoking status as covariate in multiple models. This emphasizes the importance of these risk factors in periodontal health as well as their interaction with this group of nutrients. This suggests the existence of putative risk groups to recommend the consumption of vitamins A or pro-vitamins as nutraceutical in the context of periodontal health. However, the methodological approaches of the mentioned studies makes provided evidence no definitive to consider this group of nutrients as “nutraceutical” and some additional experimental, or at least cohort, studies are needed.

The biologically active form of vitamin A, retinoic acid, is essential for maintaining the integrity of mucosal tissues and for proper differentiation of cells, including those of the immune system [28,43]. According to this, it is expected that an adequate intake of this nutrient prevent periodontal disease onset or progression since it affect to connective tissue maintenance. Moreover, its role in immune system could be also important for maintain some bacteria adequate level and for prevent over-inflammation. However, as neither indicated studies specifically evaluating vitamin A intake did not support this [36,37]. In spite of this might mean a virtual absence of relationship between this nutrient and periodontal health, it is also important to consider that none divided sample by recommended dietary amount. Consequently, if deficiencies are rare in populations where the studies were carried out, relationship could be not be elucidated when this is not linear. Moreover, other investigators have reported antioxidant activities for retinol and dehydroretinol as well as for many pro-vitamin A compounds, including *β*- and *α*-carotenes [44,45]. However, *β*-carotene is an efficient quencher of singlet oxygen [46] but vitamin A (i.e., retinol and derivatives) cannot quench singlet oxygen and has a very small capacity to scavenge free radicals [47,48]. In addition, more studies and analysis reported associations for *β*-carotene than for retinol or vitamin A. This fact would be consistent with the possibility that this effect is due to its antioxidant activity and not as vitamin A precursor. Unfortunately, there were no measurements of oxidative stress levels in the studies presented in this review.

### 2.3. Vitamin B-Complex

Vitamin B-complex refers to all of the known essential water-soluble vitamins except for vitamin C. These include thiamine (vitamin B_1)_, riboflavin (vitamin B_2_), niacin (vitamin B_3_), pantothenic acid (vitamin B_5_), pyridoxine (vitamin B6), biotin (vitamin B_7_ or B_8_), folic acid (vitamin B_9_) and cobalamin (vitamin B_12_) [49]. Four observational studies in humans, three cross-sectional studies and a case-control study met the eligibility criteria for vitamins belonging to B-complex. Concerning to experimental research there was only one report focusing on a nutritional intervention in human beings [50]. Overall, vitamin B-complex is necessary for cell growth and metabolism [49], but each member of the B-complex has a unique structure and performs unique functions. Vitamins B_1_, B_2_, B_3_, and biotin participate in different aspects of energy production, vitamin B6 is essential for amino acid metabolism, and vitamin B_12_ and folic acid facilitate steps required for cellular division [50,51,52].

The most promising results for this group of nutrients has been offered by a randomized placebo-controlled double-blind trial (Table 3) where it was tested the effect of a vitamin B-complex supplement (50 mg of B_1_, B_5_, B_2_, B_3_, and B_6_; 50 μg of B_12_ and B_7_; and 400 μg of B_9_) after access flap surgery. After 30 days of treatment, the supplement led to statistically significant superior CAL gains when compared to placebo, although PPD improved in a similar manner in all groups [50]. However, currently it is assumed that PPD alone does not reliably reflect whether teeth have progressive CAL, except in sites with deep PPD. This would support the use of vitamin B-complex supplements as nutraceutical to restore periodontal health, at least in combination with surgical interventions.

On the other hand, no associations were observed in the older investigation that analyzed clinical sings of vitamin B deficiency in relation to RPI previously indicated for vitamin A [39]. However, as mentioned, in this last periodontal disease degree could be undervalued since some oral examinations were made into bad conditions. Notwithstanding, more studies exploring the use of vitamin B-complex as nutraceutical under different conditions are needed prior to encourage its use in relation to periodontal disease.

There is little information about the separate role of the different components of this complex, although observational studies provide some information in this sense (Table 4). Among all vitamins of the B-complex, folic acid seems to be the most interesting. Actually, two of the selected cross-sectional studies for vitamin B-complex only evaluated associations with this vitamin, but when their results are combined, a clear association cannot be established. One performed with data from elderly participants from the 2001–2002 National Health and Nutrition Examination Survey (NHANES) showed that low serum folate level was independently associated with periodontal disease (defined by the presence of at least of 10% of sites with CAL of 4 mm or higher and/or PPD of at least 3 mm) [63]. In contrast, in other study based on a representative sample of adults from Japan, no association between risk of periodontal disease (defined by a CPI of 3 or more) and dietary intake estimations was found [64]. Similarly, another study that only included non-smokers subjects [65] reported no correlation of dietary intake with CPI score (i.e., periodontal disease severity) although it was inversely associated with percentage of sites with BOP. Despite low values of CPI reflect gingival bleeding and calculus, when it reach values of 3 or higher is only based on PPD and provides no information about bleeding or calculus presence. Therefore, the last study suggests that folic acid intake could be important for prevention of gingival inflammation, although the relationship with periodontal pocket formation and development is unclear. Notwithstanding, in this study only were included non-smokers individuals. In addition, they were adults belonging to any age. This could indicate that smoking and age and important risk factor interacting with vitamin B_9_, but bias associated with dietary intake assessment methods also could lead to underestimating the effect of this nutrient.

The role in periodontal disease of other vitamins belonging to B-complex has been also evaluated in an observational study but no clear association has been found for them. In the study carried out in patients from a dental clinic [36], there was no correlation between RPI (that scored the presence and severity of both gingival bleeding and pocket depth) and the dietary intakes of the three vitamins measured, thiamine, niacin and riboflavin. The same vitamins were also analyzed in the study in female adolescents from Rome already mentioned for vitamin A with results that could results interesting to clarify their roles of some vitamin B, at least at gingival inflammation level. In this case, gingivitis-affected presented lower dietary intakes of vitamins B_1_ and B_2_ with respect to those non-affected, but B_2_ was the only that remained significant in multiple logistic regression analysis. This only occurred in one of the models used in that fiber was not included, whereas in the other two that did it, Vitamin B_2_ was substituted by calcium. Authors concluded that both nutrients as strong correlated as consequence of high milk intake, so real cause for this association remain unclear [37]. Moreover, association with dietary intake of vitamin B_12_ was also evaluated in the study based on a representative sample of Japanese adults, but no significant results were reported [64]. Therefore, the paucity of studies on individual vitamins B role in periodontal health does not allow recommending supplementation with a particular vitamin B with a nutraceutical objective, although folic acid is an important candidate. In any case, the results from the RCT with B-complex supplement [50] are promising and more studies in human should be done with such combination.

### 2.4. Vitamin C

Vitamin C, also known as L-ascorbic acid, acts as enzymatic cofactor in a range of essential metabolic reactions as ascorbate [66,67]. These include hydroxylation of proline and lysine which is needed to stabilize its collagen structure during its manufacture. This activity confers to vitamin C a key role in the maintenance of the integrity of connective tissues such as the periodontium.

Actually, it is well known that vitamin C deficiency causes scurvy, but it is not certain whether milder degrees of insufficiency have clinically important effects on the periodontium. Furthermore this nutrient is involved in immunological functions, such as phagocytosis, and in wound healing. Vitamin C insufficiency may affect immunocompetence. Moreover, ascorbic acid is also known as a powerful antioxidant in living organisms, particularly at intracellular level [67]. A function in which some enzyme-dependent systems cooperate recycling oxidized forms. All these functions and activities have conferred to vitamin C an interesting potential role in periodontal health. Probably, this explains why this nutrients has been the most studied among all vitamins in relation to periodontal disease. Actually, the research into the relationship between vitamin C and periodontal-related diseases comes from as far as the eighteenth century when it was observed that scurvy was fully recovered after a treatment with oranges and lemons [68]. Since then, many experimental (Table 5) and epidemiological studies have tried to address the relationship between vitamin C and periodontal disease. In fact, a total of 14 reports have been selected for this review in relation to this topic. This makes this vitamin the most represented.

Studies in animal models have been performed on male rats [57,58], in which periodontitis was experimentally induced by placing ligature around some teeth and the effects of vitamin C-supplemented diets were compared with standard diets. Overall, results from them endorse the protective or therapeutic role of vitamin C on periodontal health. After disease development by the mentioned procedure, vitamin C showed to improve bone density index (BDI) at areas with periodontitis [58]. In relation to bone metabolism markers, the supplemented diet decreased RANKL expression suggesting that osteoclastogenesis stimulation is diminished despite an increase in alkaline phosphatase (AP) was also observed [58]. The analysis of additional markers has shown that consumption of vitamin C-supplemented diets improved the values of some oxidative stress markers (reduced glutathione (GSH) to oxidized glutathione (GSSG) ratio and 8-hydroxydeoxyguanosine (8-OhdG)) [57], decrease myeloperoxidase (MPO) activity [43] and down-regulated the expression of assessed pro-inflammatory cytokines (interleukin (IL)-1α and IL-1β) [57]; all associated to experimental periodontitis.

In regard to research in humans, 15 observational studies and six randomized-controlled trials (RCT) were found among the selected studies. Results from observational studies are contradictory and often depend on parameter evaluated. Concerning dietary intake, several studies have reported a negative association with periodontal disease. Among cross-sectional studies, one of most interesting evidences are provided by Nishida et al. [69] who reported a dose-dependent relationship between vitamin C intake and the number of persons with periodontitis (defined by an average value of CAL of 1.5 mm or higher) in a sample of 12,419 NHANES III participants. Otherwise, no relationships have been found in other investigations. These included a cross-sectional study in 80 patients from a U.S. dental clinic [21] already cited for previous vitamins and a case-control study in 42 patients from an academic center in The Netherlands [70], but also a cross-sectional study in 3043 participants from Japanese NHANES [65] where periodontitis was defined by a CPI of 3 or more. In the same sense, in the study above that compared female adolescent with and without gingivitis [37] no differences were observed in relation to prevalence of marginal deficiency of vitamin C intake.

In contrast, those studies evaluating vitamin C levels in blood mostly supported a negative association between nutritional status and periodontal disease. Among them, there was a cohort study in a younger group of Indonesian villagers reporting a negative correlation between plasma vitamin C level and CAL after 15 years [71]. Additionally, participants with vitamin C deficiency displayed higher CAL than those with no depletion or normal plasma values [71]. Likewise, case-control studies also confirmed this association [70,71]. Among them, one where authors subdivided periodontitis patients into systemically healthy subjects and those type 2 diabetes mellitus (DM2) patients [72]. However, these last results may be questioned because the report did not offer details about possible pair-matched, confounding analysis or prevention of detection bias. Interestingly, the other case-control study, cited above [70], did not find significant association with dietary intakes. In turn, in the cross-sectional study in dental clinic patients [36] did not find associations with RPI as occurred with dietary intakes. Still, vitamin C levels were negatively associated with periodontitis prevalence in other study based on the NHANES III data, previously mentioned [38]. Furthermore, this association was significant depending on periodontitis definition criteria. Despite many studies reported an absence of association between vitamin C and periodontitis, it is important to note that those based in great and representative samples did it. Moreover, there was a wide variety in these studies concerning definitions and periodontal health-related clinical parameters used, which could explain in part the differences found among their results. On the other hand, some epidemiological studies have also focused on antibody levels against certain microorganism negatively related to periodontal health. In one of this, plasma concentrations of vitamin C were inversely correlated with the number of oversensitive to *P. gingivalis* [73]. On the other hand, when total immunoglobulin G (IgG) levels to several oral bacteria were taken into account, there was no association with dietary intakes of vitamin C in a sub-cohort aged 35–70 years old from the European Prevalence of Infection in Intensive Care Study [74]. This suggest that *P. gingivalis* is particularly interesting for periodontal disease pathogenesis.

Moreover, dietary vitamin C effects in the presence of typical risk factors also have been evaluated. Among others, the possible interaction with smoking has been evaluated in studies restricted to a particular subgroup or by means of stratification. In the study by Nishida et al. [69] with NHANES III data from current and former smoker subjects, a dose-dependent relationship between vitamin C intake and periodontal disease diagnosis was reported, being stronger in current smokers. However, serum levels were negatively associated with periodontitis prevalence in other study based also on NHANES III data when only never-smokers were included, even after used a more restrictive criteria to define periodontitis [38]. Similarly, studies stratified by age or restricted to elderly people have been performed. Two cohort studies restricted to old segment of population have provided evidences for negative associations among disease and vitamin C intake. In a subset of 73 years old subjects from Niigata study, the number of teeth with periodontal disease progression for 2 years of follow-up was shown to be inversely affected by vitamin C intake, even after adjustment for potential confounder [34]. Aged subjects from the same survey showed similar associations when follow-up time was of 8 years [75]. In a cross-sectional study, elderly participants from Niigata city (Japan) exhibited a weak negative association although significant among CAL and serum vitamin C levels, even after adjustment for potential confounders in multivariate regression analysis [61]. Curiously, in a stratified cohort study in Americans that participated in the first National Health and Nutrition Examination Survey (NHANES I) and NHANES I Epidemiologic Follow-up Study (NHEFS) with a follow-up of approximately 10 years in a population of all ages, an inverse relationship with dietary intake was noted but only in younger persons (25–59 years) [76]. Therefore, although age and smoking can be related with efficacy of vitamin for periodontal disease prevention, this can depend on population features. In addition, results from a study in rats also have suggested that vitamin C supplementation could contribute to prevent the effect of other nutritional risk factors associated with this disease, namely cholesterol amount in the diet, on alveolar bone density [59]. This effect was also associated to a decrease in osteoclast differentiation markers and serum 8-OHdG initiating that it act by mechanisms related to oxidative stress and osteoclastogenesis regulation as occurred in similar models fed standard diets [57,58]. All these suggest that vitamin C also can contributed to prevent the effect of other risk factors of periodontal health the use of supplements could result particularly interesting for subjects showing such risks.

Despite evidences collected from animals and epidemiological studies, none intervention in humans presented here reported clear positive effects for vitamin C supplementation on healthy subjects or periodontitis patients. Positive effects were noted only with increases of intakes through vitamin C-rich foods introduced on diet. Main finding supporting this idea comes from a cross-over trial where the same individuals chewed on sugar free gum contained vitamin C, non-vitamin C and no gum for three months following by scaling treatment. Vitamin C-containing gum led to an improvement at gingival bleeding (GB) level, although interestingly, calculus score and visible plaque were diminished with the two gums. However, it is important to note that other clinical measurements related to periodontal disease like PPD or CAL were not taken, so treatment implications were not deeply known [53].

In relation to this parameters, another study, in this case in children, tested the effect of chewing on vitamin C enriched tablets on RPI but no effect were observed compared with mannitol-containing tablets that were used as placebo [54]. Notwithstanding, risk of performance bias was present because of variability in consumption. Likewise, no effects also was noted in other two investigations in patients that received root and planning therapy before a period of vitamin C supplementation [55,56]. However, not blinding nor detail about selection comprised results in one of them. Problems in obtaining positive results could be due to used dosages or its combination with treatment more effective [55,56] that may occult the effect of this nutrient. On the other hand, maybe correct vitamin C intake is necessary to avoid periodontal problems but when the pathological state has been instituted, a supplementation with vitamin C is not enough to revert it to the healthy state

### 2.5. Vitamin E

Vitamin E activity is exerted by a set of lipid-soluble food compounds featured by a phenolic-chromanol ring linked to an isoprenoid side chain. These are termed as tocopherols or tocotrienols depending on the phenolic-chromanol ring were saturated or unsaturated, respectively. In addition both can present four forms according to the number and position of methyl groups on the ring: α, β, γ and δ [78]. As expected, all these forms have been taken into account for this review. For this set of compounds, four observational studies in humans (Table 6) and two experimental studies in rats were found (Table 2). Regarding research in humans, there were two cohort studies and two cross-sectional studies. Serum levels of α-tocopherol have been inversely associated with mean PPD and periodontitis (defined according to CDC/AAP criteria) in a subset of participants in the 1999–2001 NHANES. However, serum levels of γ-tocopherol showed a most modest association with subjects in the interquartile range displaying a significantly lower mean PPD than those in the highest quartile [3].

The rest of observational studies were restricted to elderly subjects [41,42,77]. Two cohort studies has provided complementary data supporting the positive effect of vitamin E on the prevention of periodontal disease progression (defined by an annual loss of CAL equal or higher than 3 mm or higher at any site over the study period) but they only were performed in elderly people. One of them, that have been previously reported [41], estimated dietary intakes of vitamin E [26] and the other evaluated serum levels of α-tocopherol [77]. However, this survey could be affected by a possible detection bias due to self-assessment and no inclusion of supplement as we pointed out previously. In contrast, no association of periodontitis with α-tocopherol or γ-tocopherol levels was found in PRIME study participants [42], although it was a cross-sectional study. Anyway, both studies reporting associations were performed with subjects form Niigata city (Japan), it would be interesting evaluate this relationship in cohorts from other population to validate this association, at least in old people.

Because of their liposoluble properties, vitamin E is able to incorporate in biological membranes where it inhibits peroxidation of lipids [79,80] due to this well-known antioxidant activity [80]. As consequence, vitamin E stops the production of ROS formed when fat undergoes oxidation [78] preventing propagation of damage to cell membranes by peroxyl radicals formed by the oxidation of lipids to stabilize the membrane structure by terminating the free radical chain reaction [78,79,80]. Research in animals has allowed to evaluate the use of vitamin E supplements on periodontal diseases and its possible role on ROS and inflammatory processes associated with this conditions [46,47]. In a rat model of periodontitis induced by ligatures, vitamin E supplements prevented increases in malondialdehyde levels and immunoreactivity to inducible nitric oxide synthase (iNOS), but did not decrease ABL [61]. However, in other rats stressed on a rotational device or not stressed, supplements had a protective effect on bone loss in all cases, but there was no interaction with stress condition [62]. Therefore, experiments in rats suggest that vitamin E supplements would prevent oxidative stress associated to periodontitis [46,47], but its potential for periodontal disease treatment is not clear since they did not decreased ABL always [46]. In any case, there are no studies evaluating its possible use as nutraceutical in relation to this disease in humans.

## 3. Materials and Methods

### 3.1. Selection Criteria

Inclusion and exclusion criteria for the selection of papers to be reviewed were established prior to commencement of the literature search. For this review, researches using both humans and animals were included, but only written in English language. All relevant studies investigating the association between periodontal disease and nutrient dietary intake or nutritional status markers were included, even if they focused only on people belonging to certain age groups or on subjects with any special physiological condition or illness (pregnancy, menopause, diabetes mellitus or others). Studies had to assess periodontal health condition, including also number of teeth or edentulism. However it was considered that it could be due to other pathologies. Regarding study design, cross-sectional, cohort and case-control studies were selected within those belonging to observational type. On the other hand, only RCT were accepted from experimental surveys collected. Additionally, interventional trials with nutrient combinations were excluded for individual assessment of each nutrient, although some exceptions were made, which will be discussed later. Similar exclusion criteria were used in the case of animal studies.

### 3.2. Information Source and Search Terms

The electronic database of the National Library of Medicine (Washington, DC, USA) (MEDLINE: PubMed) was used to select appropriate papers. We first derived two themes that were then combined by using the Boolean operator ‘‘AND’’. Each theme was created by using the operator ‘‘OR’’ to search for terms appearing as either explorer text words or Medical Subjects Headings (Mesh), when they existed and it was not contained within other, also used. The selected periodontal disease related terms were: periodontal, periodontitis, gingivitis and it was also included the following outcome measurement related to periodontal disease: “Alveolar Bone Loss” [Mesh] OR “Periodontal Diseases” [Mesh] OR “Periodontal Attachment Loss” [Mesh] OR “Periodontal Index” [Mesh] OR “Gingival Hemorrhage” [Mesh] OR “periodontal disease” OR periodontitis OR “alveolar bone loss” OR “alveolar bone resorption” OR “tooth attachment” OR “tooth mobility” OR “gingivitis” OR “clinical attachment level” OR “periodontal attachment level” OR “attachment loss” OR “periodontal pocket” OR “pocket depth” OR “probing depth” OR “bleeding on probing” OR “gingival bleeding” OR “Gingival Hemorrhage” OR “gingival index” OR “bleeding index” OR “periodontal index”. In addition to this, a second theme related to nutrition or diet was created. Search terms were as follows: “Food” [Mesh] OR “Diet” [Mesh] OR “Eating” [Mesh] OR “Nutrition Surveys” [Mesh] OR “Nutrition Assessment” [Mesh] OR “Nutrition Therapy” [Mesh] OR “Nutrition Processes” [Mesh] OR “Nutritional Status” [Mesh] OR nutrition * OR nutrition OR nutrient * OR nutrient OR food OR dietary OR diet * OR intake OR intakes OR consumption* OR consumption OR ingestion OR eating. Finally, for each nutrient it was combined with all name synonyms, its precursors and its ionic forms present in organism as search terms.

### 3.3. Search Strategy

The search strategy aimed to find both published studies in the English language from the inception of the database until February 2018. A comprehensive literature search was run independently by two of the review authors. At first, papers were screened by title and abstract. Screening procedures were adjusted for higher sensitivity (with restrictive search items omitted). Secondly, full text-papers were retrieved and selected based on the eligibility criteria, and duplicated studies were excluded. Titles without abstract but which suggested that they were related to the objectives of this review were selected for full text screening to screen the full text. Inconveniently, our methodological approach did not allow to obtain articles nor available on line, or published in journals whose rights had not been acquired by University of Granada at the date of search. Review authors’ disagreements or inconsistencies concerning inclusion of publications or extraction of data were discussed to eventually achieve mutual consensus.

### 3.4. Data Collection Process, Data Items and Summary Measures

The quantitative data extracted from papers included specific details about the interventions, populations, study methods, and significant outcomes for the review question and specific objectives. Data from eligible studies were independently evaluated. When they were available, outcome measurement means or differences between means, and their standard deviations between groups like clinical outcomes for periodontal disease, nutritional status or dietary intakes, depending on study, were included in our description of the results. In the same way, association measurements, such as correlation or regression coefficients, IRRs, ORs, RRs, HRs and their corresponding 95%CI, as well as significance levels considered or *p*-values were considered, but only if significant associations and/or differences were found.

### 3.5. Quality Assessment and Risk of Bias

Subsequently, potentially relevant publications were read in full to determine their quality, mainly through the assessment of bias risk. The methodological quality of a publication was determined in accordance with several criteria depending on the study design. For observational studies, evaluation and strategies to deal with them were considered in addition to risk of bias. According to the prevention of confounding effects, the employment of randomization, only in clinical trials, matching or restriction, were explored at design level, while the use of multivariate analysis, stratification or frequency matching was appraised at data analysis level. Concerning the risk of bias, four types of bias have been proposed: selection, detection, attrition and reporting bias [81]; although other authors have simplified this classification into two categories: selection and information bias [82]. Anyway, critical issues for prevent them present differences among design types. For cross-sectional studies, three points were mainly evaluated: sample randomization, reliability and objectivity of variables assessment, as well as definition of group if comparisons were made. For case-control studies, it was also taken into account the objectivity and reliability of subjects evaluations including here category definitions and exposure evaluation methods. Lastly, for cohort studies, it was considered the exposure and status definition and measurement. In the same way, differences in duration, reliability and objectivity of outcome assessment were considered. Also, the presence of differences on intensity of medical surveillance, loss of follow-up, missing data treatment, and differences related to the outcome or exposure of risk factors between those who drop out and those who stay in the study were taken into account. Inclusion criteria and/or sources of data or individuals background were considered for the establishing of the sample external validity and set of population represented. Additionally, grouping criteria in data analysis were account, when they were presented, especially if they were selected post hoc from alternative options.

On the other hand, Intervention trials were assessed according to Cochrane guidelines, which take into account five type of bias: selection, performance, detection, attrition and reporting bias [83]. Based on their recommendations, the following domains were evaluated: sequence generation (randomization), allocation concealment, blinding of participants, operators and/or examiners, incomplete outcome data and selective reporting. In function of the results of this assessment, degree of risk (high, low or unclear) was established for each type of bias, as well as its possible magnitude and direction. The risk of bias and its possible effects were summarizing for each outcome within each study. Finally, it was summarized for review as a whole, where it was possible.

## 4. Conclusions

The aim of this review was to analyze the main published data until February 2018 on the relationship between vitamins and the development and progression of periodontal disease both in humans and animals to understand the potential benefits and risks of supplementation with vitamins on this pathology. Although some studies suggest that improving or maintaining an optimal nutritional status in relation to vitamins, particularly for vitamins D and C, in general, evidence supporting certain grade of relationship of vitamins with periodontal health is reduced and there are limitations that should be overcome. Among others, the number of experimental studies is reduced yet, particularly in humans; likewise, reported effects are usually too small to indicate the magnitude of therapeutic supplements, even when used as an adjunct to periodontal therapy. Importantly, for certain vitamins, interventions in humans did not reported clear positive effect despite evidence provided by observational and animal studies as occurred with vitamin C supplementation. Concerning observational studies, the use of dietary supplement was ignored or normalized with the same value for all subjects in some of them. Moreover, there was a wide variety in observational studies concerning definitions and periodontal health-related clinical parameters used, which could explain in part the differences found among their results. In addition to that, few articles deepened on the mechanisms under the observed relationships. On the other hand, several studies have been performed on subgroups with conditions that represent a risk for bone health such as age, menopause or smoking status. The use of supplements could result particularly interesting for subjects showing such risks. For instance, different studies on vitamin D suggest that starting to take vitamin D supplementation from middle age could be particularly interesting for preventing periodontal disease advancement.

Problems in obtaining positive results from experimental studies could be due to used dosages or its combination with treatment more effective that may occult the effect of this nutrient. In spite of this, there is a paucity of well-designed, long-term experimental studies to ascertain the direct effects of dietary supplements on the outcomes on periodontal diseases. Therefore, the results and methodological approaches of many of the presented studies makes provided evidence no definitive to clearly consider as “nutraceutical” any of the vitamins included in this review. In this sense, is particularly important to note that supplementation is not enough to revert the periodontium to a healthy state, or at least at the moment there is not enough evidence supporting this. Therefore, the use of vitamin supplements in any case can replace other therapies. Notwithstanding, the use of supplements with several or certain vitamins for people under physiological or pathological conditions that increase the risk of suffers bone pathologies or pathological alterations could contribute to protect periodontal tissues. This suggests the existence of putative risk groups to recommend the consumption of nutraceutical in the context of periodontal health, but more studies are needed to confirm this and clarify what vitamins use for each case, as well as dosages and taking frequency.

## Figures and Tables

**Table 1:** Observational studies on vitamin D association with periodontal disease.

Study Type	Sample	Sex; Age; N	Dietary Intake Assessment	Nutritional Status Assessment	Periodonta Status	Analysis Results (*p*-Value)	Main Results/Conclusions	Ref.
CS	DANHES 2007–2008 participants (Denmark)	Both; ≥18 y; N = 3287	Vitamin D intake by FFQ	-	Severe periodontitis ^1^	N.S. (mult. logistic reg.)	No association	[30]
					CAL	N.S. (mult. linear reg.)		
CS	DLS participants (USA)	Male; 62.9 ± 7.6 y; N = 562	Daily dietary intake (≥400 IU, 400–800 IU or ≥800 IU) by FFQ	-	Severe periodontal disease ^1^	OR ^1^ = 0.67 (95%CI: 0.55–0.81); *p* < 0.05 for trend (mult. logistic reg.)	Inverse associations	[28]
					Moderate to severe ABL ^2^	OR ^1^ = 0.54 (95%CI: 0.30–0.96); *p* < 0.05 for trend (mult. logistic reg.)		
CS	NHANES III participants (USA)	Both; ≥20 y; N = 11,202	-	Serum levels of 25(OH)D (quintile)	Mean CAL	N.S. (mult. linerar reg.) in men <50 y old	Negative association with CAL, only in subjects ≥50 years	[27]
						CAL ^2^ = +0.39 mm (95%CI: 0.17–0.60); *p* = 0.001 (mult. linerar reg.) in men ≥50 y old		
						N.S. (mult. linerar reg.) in women <50 y old		
						CAL ^2^ = +0.26 mm (95%CI: 0.09–0.43); *p* = 0.008 (mult. linerar reg.) in women ≥50 y old		
					% BOP	N.S. (mult. linerar reg.) in all subsets		
CS	4th KNHANES Participants (South Korea)	Both; ≥19 y; N = 6011	-	Serum levels of 25(OH)D (>20, <20 ng/mL) deficiency	CPI ≥ 3	N.S. (mult. logistic reg.) in non-restricted model	Inverse association only in current smokers	[31]
						OR ^2^ = 1.53 (95%CI: 1.07–2.18) (mult. logistic reg.) in model resticted to current smokers		
						N.S. (mult. logistic reg.) in model restricted to non-current smokers		
CS	Paticipants in OsteoPerio Study (USA)	Female, Postmenopause; N = 920	-	Plasma levels of 25(OH)D (Adequate or inadequate nutritional status)	50% BOP	OR = 0.42% (95%CI: 21–58%); *p* < 0.05 for trend	Inverse association with % BOP & periodontal disease according to CDC/AAP criteria ^5^	[33]
					periodontal disease based on ACH measures ^3^	N.S.		
					periodontal disease based on CDC/AAP criteria ^1^	OR = 33% (95%CI: 5–53%) *p* < 0.05 for trend		
					tooth loss	N.S.		
CC	Subjects from an University Health Center (China)	Both; 16–64 y; N = 178	-	Plasma levels of 25(OH)D	Aggressive periodontitis ^4^ vs. chronic periodontitis-affected ^4^ vs. healthy (Staff)	29.28 in aggressive periodontitis patients >21.60 nmol/L in controls; *p* < 0.05 (ANCOVA)	Plasma 25(OH)D levels in patients with aggressive periodontitis were higher than those of healthy controls	[35]
CC	Women with a singleton pregnancy taking vitamin D supplements (USA)	27 ± 6 for cases/31 ± 6 y for controls; N = 235	-	Serum levels of 25(OH)D	Clinical moderate to severe periodontal disease-affected ^5^ vs. healthy	59 > 100 nmol/L; *p* < 0.001 (Wilcoxon Mann-Whitney U)	Subject with periodontal disease had lower serum levels of 25(OH)D	[34]
				Women with vitamin D deficiency (<75 nmol/L)		65 > 29%; *p* < 0.001) (x^2^ Test). Moreover, multivariable analysis showed an adjusted OR of 2.1 between periodontal disease and vitamin D insufficiency (95%CI. 0.99–4.5)		
CC	Cases of moderate/severe periodontitis & periodontally healthy controls aged. (Puerto Rico, USA)	Both; 35–64 y; N = 38	-	Serum levels of 25 (OH) D levels	periodontal disease	18.5 (± 4.6) ng/mL < 24.2 (± 7.1) ng/mL; *p *= 0.006.	Inverse association	[29]
				Serum levels of 25 (OH) D levels		OR = 0.885 (95%CI: 0.785–0.997) *p *< 0.05 for trend		
C (5 y)	Postmenopausal paticipants in OsteoPerio Study (USA)	Female; Postmenopause; N = 665	-	Plasma levels of 25(OH)D	Mean ACH increase (1 mm)	N.S. (mult. linear reg.)	No association	[32]
					Mean CAL increase (1 mm)	N.S. (mult. linear reg.)		
					Mean PD increase (1 mm)	N.S. (mult. linear reg.)		
					Mean % BOP increase (1%)	N.S. (mult. linear reg.).		
C (20 y)	Health Professionals Follow-Up Study participants (USA)	Both; 40–75 y; N = 42,730	Semi-quantitative FFQ	Predicted 25(OH)D score ^6^ (quintiles)	Tooth loss incidence	HR = 0.90 (95%CI: 0.88–0.92); *p* < 0.001 for trend (COX model)	Inverse dose-dependent associations	[26]
					Self-reported periodontitis	HR = 0.91 (95%CI: 0.86–0.95); *p* < 0.001 for trend (COX model)		

^1^ values compared highest vs. lowest percentile, ^2^ values compared lowest vs. highest percentile, ^1^ AAP/CDC criteria: PPD ≥ 5 mm on at least 1 tooth and CAL ≥ 6 mm at 2 or more sites (not on same tooth), ^2^ ABL ≥ 40% at 3 or more sites. ^3^ ABL ≥ 40% or ≥1 tooth lost, ^4^ IWCPDC criteria: ^3^ BOP & PPD ≥ 3 mm at ≥1 sites. AL > 2 mm or ≥1 tooth lost, ^5^ ≥15 sites with PPD ≥4 mm. Abbreviations: 95%CI: 95% confidence interval, 25(OH)D: 25-hydroxyvitamin D, AAP: American Academy of Periodontology, ABL: alveolar bone loss, ACH: alveolar crestal height, BOP: bleeding on probing, CAL: clinical attachment loss, CC: case-control study, CDC: Center for Disease Control and Prevention, CS: cross-sectional study, DLS: Department of Veteran Affairs Dental Longitudinal Study, HR: hazard ratio, IWCPDC: International Workshop for the Classification of Periodontal Diseases & Conditions in 1999, NHANES III: Third National Health and Nutrition Examination Survey, OR: odds ratio, PPD: periodontal probing pocket, US: United States, vs.: versus, w: weeks.

**Table 2:** Observational studies on association of vitamin and provitamins A with periodontal disease.

Study Type	Sample	Sex; Age; N	Dietary Intake Assessment	Nutritional Status Assessment	Periodontal Status	Analysis Results (*p*-Value)	Main Results/Conclusions	Ref
CS	Dental clinic patients (USA)	Both; N.A.; N = 80	Dietary intakes of vitamin A by 24-h recall (N = 56)	Serum levels of vitamin A	RPI	N.S. (mult. linear reg with R^2^ improvement)	No associations	[36]
CS	Ethnic group subjects (Sri Lanka)	Both; ≥13 y; N = 7944	-	Vitamin A deficiency determined by clinical symptoms	RPI	N.S. (mult. linear reg.)	No association	[39]
CS	NHANES III participants (USA)	Both; ≥20 y; N = 11,480	-	Serum levels of *α*-carotene (quintiles)	Mild periodontitis ^3^	OR ^1^ = 0.60 (95%CI: 0.46–0.77); *p* = 0.0009 for trend (mult. logistic reg.) in non-restricted model	Negative association of β-& α-carotene, & β-Cryptoxanthin with mild periodontitis; & of β-carotene & vitamin A with severe periodontitis	[38]
						N.S. (mult. logistic reg.) in model restricted to never-smokers		
					Severe periodontitis ^4^	N.S. (mult. logistic reg.) in both models		
				Serum levels of β-carotene (quintiles)	Mild periodontitis ^3^	OR ^1^ = 0.80 (95%CI: 0.65, 0.98); *p* = 0.0001 for trend (mult. logistic reg.) in non-restricted model		
						OR ^1^ = 0.99 (95%CI: 0.73–1.35); *p* = 0.044 for trend (mult. logistic reg.) in model restricted to never-smokers		
					Severe periodontitis ^4^	OR ^1^ = 0.65 (95%CI: 0.46–0.93); *p* = 0.001 (mult. logistic reg.) in non-restricted model		
						N.S. (mult. logistic reg.) in model restricted to never-smokers		
				Serum levels of β-cryptoxanthin (quintiles)	Mild periodontitis ^3^	OR ^2^ = 0.74 (95%CI: 0.61–0.89) *p* = 0.03 for trend (mult. logistic reg.) in non-restricted model		
						N.S. (mult. logistic reg.) in model restricted to never-smokers		
					Severe periodontitis ^4^	N.S. (mult. logistic reg.) in both models		
				Serum levels of vitamin A (quintiles)	Mild periodontitis ^3^	N.S. (mult. logistic reg.) in both models		
					Severe periodontitis ^4^	OR ^1^ = 0.77 (95%CI: 0.58–1.03) *p* = 0.049 for trend (mult. logistic reg.) in non-restricted model		
						N.S. (mult. logistic reg.) in model restricted to never-smokers		
CS	Participants in PRIME (Northern Ireland)	Male; 60–70 y; N = 1258	-	Serum levels of retinol (quintiles)	Low-threshold periodontitis ^5^	OR ^2^ = 1.64 (95%CI: 1.07–2.51); *p* = 0.008 for trend (mult. logistic reg.)	Negative association of serum levels of β-carotene & β-cryptoxanthin at both tresholds, & also of α-carotene only at low-threshold	[42]
					High-threshold periodontitis ^6^	N.S. (mult. logistic reg.)		
				Serum levels of *α*-carotene (quintiles)	Low-threshold periodontitis ^5^	OR ^2^ = 1.81 (95%CI: 1.17–2.82); *p* < 0.001 for trend (mult. logistic reg.)		
					High-threshold periodontitis ^6^	N.S. (mult. logistic reg.)		
				Serum levels of β-carotene (quintiles)	Low-thresholdperiodontitis ^5^	OR ^2^ = 1.83 (95%CI: 1.19–2.80); *p* < 0.001 for trend (mult. logistic reg.)		
					High-threshold periodontitis ^6^	OR ^2^ = 1.87 (95%CI: 0.86–4.06); *p* = 0.01 for trend (mult. logistic reg.)		
				Serum levels of β-cryptoxanthin (quintiles)	Low-threshold periodontitis ^5^	OR ^2^ = 1.50; 95%CI: 0.99–2.27); *p* = 0.02 for trend (mult. logistic reg.)		
					High-threshold periodontitis ^6^	OR ^2^ = 4.02 (95%CI: 1.61–9.99); *p* = 0.001 for trend (mult. logistic reg.)		
CC	Non-smokers adolescents (Italy)	Female; 17–19 y; N = 54	Dietary intake of vitamin A by 3-d record	-	Gingivitis affected ^7^ vs. non-affected	N.S. differences between groups (Student *t* test)	No association	[37]
			Dietary intake marginal deficiency (<1/3 RDA) of vitamin A by 3-d record			N.S. (*X*^2^ test with Yates correction)		
RC (2 y)	Niigata study participants (Japan)	Both; 75 y; N = 334	Dietary intakes of α-carotene (tertiles) by BDHQ	-	No. of teeth with periodontal disease progression ^8^	N.S. (mult. logistic reg.)	Negative association with β-carotene intake	[41]
			Dietary intakes of β-carotene (tertiles) by BDHQ			IRR ^1^ = 0.73 (95%CI: 0.56–0.95); *p* < 0.05 for trend (mult. logistic reg.)		

^1^ values compared highest vs. lowest percentile, ^2^ values compared lowest t vs. highest percentile, ^3^ at least 1 site with CAL ≥4 mm & PPD ≥4 mm, ^4^ ≥2 mesiobuccal sites with CAL ≥5 mm & 1 mesiobuccal sites with PPD ≥4 mm, ^5^ at least 2 teeth with non-contiguous inter-proximal sites with CAL ≥6 mm & 1 PPD of ≥5 mm, ^6^ ≥15% of all sites measured CAL ≥6 mm & there was at least one site with PPD ≥6 mm, ^7^ ≥1 site with BOP, ^8^ CAL ≥ 3 mm in ≥1 site. Abbreviations: 95%CI: 95% confidence interval, BDHQ: brief diet-history questionnaire, BOP: Bleeding on probing; C: Cohort study, CAL: Clinical attachment loss, CPI: Community periodontal index; CS: Cross-sectional study; d: days, h: hours, IRR: incidence rate ratio, mult.: multiple, N: sample size, N.S.: not significant, NHANES III: Third National Health and Nutrition Examination Survey, OR: Odds ratio, PRIME: Prospective Epidemiological Study of Myocardial Infarction, PPD: periodontal pocket depth, RC: retrospective cohort study, RDA: recommended dietary amount, Ref: reference, reg.: regression, RPI: Russell’s Periodontal Index, SD: Standard deviation; US: United states, vs.: versus, y: years.

**Table 3:** Experimental studies on vitamin effects on periodontal disease.

Subjects/Animals; Age; N	Experimental Design (Duration)	Results Data (*p*-Value)	Main Results/Conclusions	Ref.
	RCT (DB) B-complex supplement for 30 d (50 mg of B_1_, B_5_, B_2_, B_3_, and B_6_; 50 μg of B_12_ and B_7_; and 400 μg of B_9_) after access flap surgery (180 d)	ΔCAL: 0.41 > −0.52 mm (*p* = 0.024)	B-complex led to superior CAL gains when compared to placebo whereas PPD improved in similar manner among groups	[50]
		ΔPPD: −1.50 ± 0.21 = −1.57 ± 0.34 mm (N.S.)		
Calculus formers from Staff & patients from a Faculty of Odontology (Sweden); Age not provided; N = 60	Chew on 0 or 5 sugar free gum/day contained 60 mg or 0 mg vitamin C (CO) (3 m per treatment)	Calculus index, Erosion scores, GB, visible PI, & saliva secretion rates (*p* < 0.05)	Vitamin C & non-vitamin gum reduced visible plaque, but No. of bleeding sites only with the first one	[53]
Children with ≤1 fully erupted tooth following routine prophylaxis; 5–20 y; N = 267	Chew on tablets containing vitamin C or mannitol (DB) (28 d)	RPI (N.S.) Teeth lost (N.S.)	No effect	[54]
Chronic periodontitis patients with ≤20 teeth receiving non-surgical treatment & health Subjects (Syria); 23–65 y; N = 60	Adjunctive dose of vitamin C or none (4 w after non-surgical treatment)	Plasma TAOC, PPD, CAL, % BOP, PI & GI	Non-surgical treatment increases plasma TAOC & improves clinical measures, but vitamin C supplements have no effect	[55]
Patients with vary degrees of gingivitis (USA); 25–60 y; N = 41	Vitamin C-supplemented or non-supplemented diet (14 d), after receiving scaling & curettage therapy in one quadrant or not	Levels of vitamin C in blood & gingiva, microscopy evaluation of gingival biopsies	Vitamin C supplementation increased Vitamin C levels in blood	[56]
Male Wistar rats; 8 w; N = 35	Ligatures placement or not placement (6 w) combined or not with vitamin C supplementation (last 2 w)	Plasma levels of vitamin C & ROM, gingival GSH/GSSG & levels of 8-OhdG Genetic expresion study in gums ^1^	Vitamin C increased plasma vitamin C level, improved GSH/GSSG & decreased 8-OHdG level (61% lower) & down-regulated some inflammation genes expression (*p* < 0.01)	[57]
Male Wistar rats; N.A.; N = 36	Ligatures placement or not placement (7 w) combined or not with supplemens of vitamin C (last 2 w)	Serum levels of BAP, gingival MPO activity, RANKL expression, BDI in mandible	Either supplement led to lower MPO activity, & RANKL expression, but higher AP; & improved BDI at the periodontitis areas	[58]
Male Wistar rats; 8 w; N = 24	1 or 2 folds vitamin C-supplemented high-cholesterol diet, non-supplemented, or standard diet (12 w)	Alveolar BMD, TRAP-positive osteoclastic cells & 8-OHdG level in periodontal tissues 8-OHdG (1663 & 1675 mg/cm in vitamin C groups vs. 1430 (−80) mg/cm in cholesterol group, *p* < 0.001 and vs. 1556 mg/cm in control groups, *p* < 0.05), osteoclast differentiation (1.1 & 0.8 vs. 4.2 No. of tartrate-resistant acid phosphatase [TRAP]-positive osteoclasts/0.1 mm, *p* < 0.05) &decreased serum 8-OHdG expression (0.12 & 0.10 vs. 0.14 ng/mL, *p* < 0.05)	Vitamin C supplements reduced the effect of high-cholesterol diet on BMD, osteoclast differentiation & decreased 8-OhdG and osteoclast differentiation kit	[59]
Male rats with non-osteogenic hereditary disorder (ODS od/od) & without it (ODS +/+); 5 w; N = 28	Vitamin C-deficient, minimally supplemented or sufficient supplemented diet in non-osteogenic disorder group (4 w)	Plasma level of vitamin C, histological observations of periodontium, BMD & cephalometric evaluation	Vitamin C deficiency influenced periodontal ligament & craniofacial growth	[60]
Male Wistar rats; 180–220 g; N = 72	Vitamin E supplements or placebo; combined with ligatures placement or sham-operations (9 d)	ABL & malondialdehyde formation, SOD activity, iNOS & TNF-α levels (1.89 ± 0.35 vs. 3.13 ± 0.42 mM of malondialdehyde, *p* < 0.01)	Vitamin E prevented malondialdehyde formation & reduced the immunoreactivity to iNOS levels	[61]
Male rice rats; 92–119 d; N = 32	Vitamin E supplemented or non-supplemented diet (122 d), combined with stress by a rotational device (last 90 d)	ABL (0.369 ± 0.091 vs. 0.436 ± 0.036 mm of CEJ-ABC, *p* = 0.017),	Vitamin E had a protective effect on bone loss which was most pronounced at sites most susceptible to loss	[62]

^1^ Genetic expression analysis for 86 genes implicated on: oxidative or metabolic stress, heat shock, proliferation and carcinogenesis, growth arrest and senescence, and necrosis or apoptosis. Abbreviations: 8-OHdG: 8-hydroxydeoxyguanosine, ABL: alveolar bone loss; BAP: bone alkaline phosphatase, BDI: Bone Density Index, BI: Bleeding index, BMD: Bone mass density, BOP: Bleeding on probing, CAL: Clinical attachment level, CO: Cross-over, d: days, DB: Double-bind, GB: gingival bleeding, GI: Gingival index, GSH/GSSG: reduced glutathione to oxidized glutathione ratio, IL: Interleukin, iNOS: Inducible nitric oxide synthase, m: moth, MPO: Myeloperoxidase, ODS: Osteogenic disorder Shionogi, PI: Plaque index, PPD: Periodontal probing depth, RANKL: Receptor activator of nuclear factor κB ligand, RCT: Randomized controlled trial, ROM: Reactive oxygen metabolites, RPI: Russell’s periodontal index, SOD: Superoxide dismutase, TAOC: total antioxidant capacity, TNF-α: Tumor necrosis factor alpha, TRAP: Tartrate-resistant acid phosphatase, US: United Sates of America, w: weeks, y: years.

**Table 4:** Observational studies on vitamin B-complex association with periodontal disease.

Study Type	Sample	Sex; Age; N	Dietary Intake Assessment	Nutritional Status Assessment	Periodontal Status	Analysis Results (*p*-Value)	Main Results/Conclusions	Ref
CS	NHANES 2001/02 Participants (US)	Both; ≥60 y; N = 844	-	Serum levels of vitamin B_9_ (quartiles)	Periodontal disease ^2^	OR ^1^ = 0.28 (95%CI: 0.30–1); *p* = 0.03 for trend (mult. logistic reg.)	Negative association	[63]
CS	Non-smokers adults (Japan)	Both; ≥18 y; N = 497	Dietary intake of vitamin B_9_	-	CPI	N.S. (mult. logistic reg.)	Negative association with % BOP	[64]
					% BOP	β= −0.204; *p* < 0.001 (mult. logistic reg.)		
CS	NHANES participants (Japan)	Both; ≥20 y; N = 3043	Dietary intake of vitamins B_9_ & B_12_ by FFQ	-	CPI = 3–4	N.S. (mult. logistic reg.)	No associations	[65]
CS	Dental clinic patients (US)	Both; N/A; N = 80	Dietary intakes of vitamins B_1_, B_2_ & B_3_ (N = 56)	-	RPI	N.S. (mult. linear reg. with R^2^ improvement)	No associations	[36]
CS	Subjects from an ethnic group (Sri Lanka)	Both; ≥13 y; N = 7944	-	Vitamin B deficiency determined by clinical symptoms	RPI	N.S. (mult. linear reg.)	No association	[39]
CC	Non-smokers adolescents (Italy)	Female; 17–19 y; N = 54	Dietary intakes of vitamin B_1_ by 3-d record	-	Gingivitis affected ^3^ vs. non-affected	0.6 (±0.2) < 0.9 (±0.3) mg/d; *p* = 0.001 (Student *t* test)	Negative association with vitamin B_2_ intake	[37]
						N.S. (Stepwise Mult. logistic reg.)		
			Dietary intakes of vitamin B_2_ by 3-d record			1.1 (±0.3) < 1.4 (±0.3) mg/d; *p* = 0.0009 (Student *t* test)		
						OR = 0.06 (96%CI: 0.01–0.35); *p* = 0.002 (Stepwise Mult. logistic reg.)		
			Dietary intakes of vitamin B_3_ by 3-d record			N.S. (Student *t* test)		
			Dietary intakes marginal deficiency (<2/3 RDA) of vitamin B_1_ by 3-d record			51.9 > 22.2%; *p* = 0.04 (*X*^2^ test with Yates correction)		
			Dietary intakes marginal deficiency (<2/3 RDA) of vitamin B_2_ by 3-d record			29.6 > 0% *p* = 0.007 (*X*^2^ test with Yates correction)		
			Dietary intakes marginal deficiency (<2/3 RDA) of vitamin B_3_ by 3-d record			N.S. *(X*^2^ test with Yates correction)		

^1^ values compared highest vs. lowest percentile, ^2^ ≥10% sites with CAL >4 mm & ≥10% sites PPD >3 mm, ^3^ ≥1 site with BOP. Abbreviations: 95%CI: 95% confidence interval, BOP: bleeding on probing; CC: Case-control study, CPI: Community periodontal index, CS: cross-sectional study, d: days, mult.: multiple, N: sample size, N.S.: not significant, NHANES: National Health and Nutrition examination Survey, OR: Odds ratio, Ref: reference, reg.: regression, RPI: Russel’s Periodontal Index, RDA: recommended dietary amount, US: United States, vs.: versus, y: years.

**Table 5:** Observational studies on vitamin C association with periodontal disease.

Study Type	Sample	Sex; Age; N	Dietary Intake Assessment	Nutritional Status Assessment	Periodontal Status	Analysis Results (*p*-Value)	Main Results/Conclusions	Ref
CS	NHANES III Participants (US)	Both; ≤20 y; N = 12,419	Dietary intake by 24-h recall (0–29, 30–59, 60–99, 100–179, & ≥180 mg)	-	Periodontal disease ^3^	OR ^1^ = 1.28 (95%CI: 1.02–1.43); *p* < 0.05 for trend (mult. logistic reg.) in current smokers	Negative association in smokers, it was stronger in former smokers than current tobacco users	[69]
						OR ^1^ = 1.21 (95%CI: 102–1.43); *p* < 0.05 for trend (mult. logistic reg.) in former smokers		
CS	NHANES III participants (US)	Both; ≤20 y; N = 11,480	-	Serum levels (quintiles)	Mild periodontitis ^4^	OR ^2^= 0.82 (95%CI: 0.76–0.87); *p* = 0.0001 (mult. logistic reg.) in non-restricted model	Negative association, it was stronger with severe disease, & in never-smokers	[38]
						OR ^2^= 0.71; (95%CI: 0.58–0.86); *p* = 0.001 for trend (mult. logistic reg.) in model restricted to never-smokers		
					Severe periodontitis ^5^	OR ^2^ = 0.76 (95%CI: 0.69–0.84); *p* = 0.0001 for trend) in non-restricted model		
						OR ^2^= 0.8 (95%CI: 0.71–0.89); *p* = 0.0001 for trend (mult. logistic reg.) in model restricted to never-smokers		
CS	Niigata study participants (Japan)	Both; 70 y; N = 413	-	Serum levels	CAL	β = −0.04 (95%CI: −0.06 to −0.005); *p* < 0.05 (mult. linear reg.)	Negative association	[77]
CS	Dental clinic patients (US)	Both; N.A.; N = 80	Dietary intakes by 24-h recall	Serum levels	RPI	N.S. (mult. linear reg with R^2^ improvement)	No associations	[36]
CS	NHANES participants (Japan)	Both; ≥20 y; N = 3043	Dietary intake by 24-h recall	-	CPI = 3–4	N.S. (mult. logistic reg.)	No associations	[65]
CS	Men from 2 populations (Finland & Russia)	Male; 48.2 ± 13.6/44.8 ± 11.4 y; N = 431	-	Plasma levels	Serum IgG against Pg	r = −0.22; *p* < 0.001 (Pearson correlation analysis)	Negative association with anti-Pg IgGs level	[73]
					Serum IgG against Agact	N.S. (Pearson correlation analysis)		
CS	Cancer-free individuals from an EPIC sub-cohort (Europe)	Both; 35–70 y; N = 395	Dietary intake *	-	Total IgGs levels for 25 oral bacteria ^6^	N.S. (generalized linear models)	No association	[74]
CC	Non-smokers outpatients (India)	Both; 30–60 y; N = 60	-	Serum levels	DM2 & periodontal disease-affected7 only periodontal disease-affected & healthy	0.93 (±0.24) > 0.335 (±0.5) > 0.292 (±0.004); *p* < 0.001 (student *t* test)	Subjects with periodontal disease showed lower vitamin C levels	[72]
CC	Patients from an academic center (NL)	Both; ≥21 y; N = 42	-	Levels in plasma	Periodontitis-affected ^8^ vs. healthy	8.3 (±3.9) < 11.3 (±5.2) mg/L; *p* = 0.03 (N.A.)	Subjects with periodontitis showed lower plasma levels of vitamin C	[70]
			No. of servings (of ≥31, 30 to 2, or <2 mg per 100 mg) by 3-d record			N.S. differences		
			Total intake by 3-d record			N.S. differences		
CC	Non-smokerss adolescents (Italy	Female; 17–19 y; N = 56	Dietary intake by 3-d record	-	Gingivitis affected ^9^ vs. non-affected	N.S. differences (Student *t* test)	No association	[37]
			Dietary intakes marginal deficiency (<2/3 RDA) by 3-d record			N.S. differences (*X*^2^ test)		
RC (2 y)	Niigata study participants (Japan)	Both; 73 y; N = 264	Dietary intake (tertiles) by BDHQ	-	No. of teeth with periodontal disease progression ^10^	IRR ^2^ = 0.72 (95%CI: 0.56–0.93); *p* < 0.05 for trend (mult. logistic reg.)	Negative association	[41]
C (8 y)	Niigata study participants (Japan)	Both; 71 y; N = 224	-	Serum levels (tertiles)	Periodontal disease events ^11^	RR ^2^ = 1.30 (95%CI: 1.16–1.47); *p* < 0.05 for trend (mult. logistic reg.)	Negative association	[77]
RC (3 y)	Residents from a village (Indonesia)	Both; 33–43 y; N = 123	-	Plasma levels (>2; 2–3.9; or ≥4.0 mg/L)	Mean CAL	β = −0.199; *p* = 0.029 (mult. linear reg.)	Negative association	[72]
C (10 y)	NHEFS & NHANES I participants (US)	Both; 25–74 y; N = 10,523	Dietary intake (% NHANES standards) by 24-h recall & FFQ	-	Total teeth lost	β = −0.0031 (±0.0011); *p* = 0.01 (mult. linear reg.) in 25–59 y old subjects	Negative association only in younger subjects	[76]
						N.S. (mult. linear reg.) in 60–74 y old subjects		

^1^ values compared lowest vs. highest percentile, ^2^ values compared highest vs. lowest percentile, ^3^ mean CAL ≥1.5 mm, ^4^ ≥1 site with CAL ≥4 mm & PPD ≥4 mm, ^5^ ≥2 mesiobuccal sites with CAL ≥5 mm & 1 mesiobuccal sites with PPD ≥4 mm, ^6^ Pg ATCC 33277 & ATCC 53978, Agact ATCC 29523 & ATCC 43718, *Tannerella forsythia* ATCC 43037, *Fusobacterium nucleatum* ATCC 25586, *Fusobacterium periodontium* ATCC 33693, *Fusobacterium polymorphum* ATCC 10953, Prevotella intermedia ATCC 25611, Prevotella melaninogenica ATCC 25845, Prevotella nigrescens ATCC 33563, Veillonella atypica ATCC 17744, *Veillonella parvula* ATCC 10790, *Bifidobacterium dentium* ATCC 27534, *Corynebacterium matruchotii* ATCC 14266, *Enterococcus faecalis* ATCC 29212, *Parvimonas micra* ATCC 33270, *Peptostreptococcus anaerobius* ATCC 27337, *Streptococcus intermedius* ATCC 27335, *Streptococcus mitis* ATCC 49456 & *Streptococcus salivarius* ATCC 7073, ^7^ generalized CAL ≥5 mm & BOP, ^8^ ABL >1/3 of the root length in >1 tooth per quadrant, ^9^ ≥1 site with BOP, ^10^ CAL ≥3 mm in ≥1 site, ^11^ No. of teeth with a loss of CAL ≥3 mm. Abbreviations: 95%CI: 95% confidence interval, Agact: *Aggregatibacter actinomycetemcomitans*, BOP: Bleeding on probing; C: Cohort study, CAL: Clinical attachment level, CC: Case-control study, CEJ: Cementum-emanel junction, CS: cross-sectional study, EPIC: European Prospective Investigation into Cancer and Nutrition, DM1: Diabetes mellitus type 1, DM2: Diabetes mellitus type 2, FBG: Fasting blood glucose, FFQ: food frequency questionnaire, IgG: Immunoglobulin G, IRR: Incidence rate ratio, IWCPDC: International Workshop for the Classification of Periodontal Diseases & Conditions in 1999, NHANES I: First National Health and Nutrition Examination Survey, NHANES III: Third National Health and Nutrition Examination Survey; NHEFS: NHANES I Epidemiologic Follow-up Stud, NL: Netherlands, OR: Odds ratio; Pg: Porphyromonas gingivalis, PBMC: Peripheral blood mononuclear cells, PI: Plaque index; PMN: Polymorphonuclear neutrophils, PPD: Periodontal probing depth; RBG: Random blood glucose, Russell’s periodontal index; RR: Relative risk, US: Unite States, vs.: versus, y: years. * by extensive quantitative dietary questionnaires or semi-quantitative FFQ or combined food records & questionnaires quartiles.

**Table 6:** Observational studies on vitamin E association with periodontal disease.

Study Type	Sample	Sex; Age; N	Dietary Intake Assessment	Nutritional Status Assessment	Periodontal Status	Analysis Results (*p*-Value)	Main Results/Conclusions	Ref
CS	1999–2001 NHANES participants (US)	Both; adults N = 4708	-	Serum levels of α-tocopherol adjusted by cholesterol levels (quartiles)	mean PPD	Mean ^1^ = 1.07 (95%CI: 1.00–1.15); 0.96 (95%CI: 0.90, 1.02) *p* = 0.005 for trend (mult. logistic reg.)	Non-linear inverse association of α-tocopherol levels with mean PPD & periodontitis	[3]
					CAL	N.S. (mult.logistic reg.)		
					Periodontitis ^3^	OR^1^ = 1.65 (95%CI: 1.26–2.16); *p* = 0.005 for trend (mult. logistic reg.)		
				Serum levels of γ-tocopherol adjusted by cholesterol levels (quartiles)	CAL	N.S. (mult.logistic reg.)		
					mean PPD	N.S. (mult.logistic reg.)		
					Periodontitis ^3^	N.S. (mult.logistic reg.)		
CS	PRIME participants (Northern Ireland)	Male; 60–70 y; N = 1258	-	Serum levels of α-tocopherol & γ-tocopherol (quintiles)	Low-threshold ^4^ Periodontitis	N.S. (mult.logistic reg.)	No associations	[42]
					High-threshold ^5^ periodontitis	N.S. (mult.logistic reg.)		
C (8 y)	Niigata study participants (Japan)	Both; 71 y; N = 224	-	Serum levels of α-tocopherol (tertiles)	Periodontal disease events ^6^	RR ^2^ = 1.15 (95%CI: 1.04–1.28); *p* < 0.05 for trend (mult.logistic. reg.)	Negative association	[75]
RC (2 y)	Niigata study participants (Japan)	Both; 75 y; N = 264	Dietary intakes of vitamin E (tertiles) by BDHQ	-	No. of teeth with periodontal disease progression ^7^	IRR ^2^ = 0.55 (95%CI: 0.42–0.72); *p* < 0.005 for trend (mult. logistic reg.)	Negative association	[42]

^1^ values compared lowest vs. highest percentile, ^2^ values compared highest vs. lowest percentile, ^3^ sum of mild, moderate, and severe periodontitis according to CDC/American Academy of Periodontology criteria, ^4^ ≥2 teeth with non-contiguous inter-proximal sites with CAL ≥6 mm & 1 PPD ≥5 mm, ^5^ ≥15% of all sites measured CAL ≥6 mm & ≥1 site with PPD ≥6 mm, ^6^ No. of teeth with CAL ≥3 mm, ^7^ CAL ≥3 mm in one site. Abbreviations: 95%CI: 95% confidence interval, BDHQ: brief diet-history questionnaire, C: cohort study, CAL: clinical attachment loss, CS: cross-sectional study, IRR: incidence rate ratio, mult.: multiple, N: sample size, N.S.: not significant, OR: odds ratio, PPD: preiodontal probing depth, PRIME: Prospective Epidemiological Study of Myocardial Infarction, reg.: regression, RR: relative risk, US: United States, y: years.
